# In Loving Hands: How Founders’ Affective Commitment Strengthens the Effect of Organizational Flexibility on Firms’ Opportunity Exploitation and Performance

**DOI:** 10.3389/fpsyg.2021.623847

**Published:** 2022-02-17

**Authors:** Christopher Pryor, Chang Li, Anastasia V. Sergeeva, Iana S. Pryor

**Affiliations:** ^1^Warrington College of Business, University of Florida, Gainesville, FL, United States; ^2^School of Business Administration, Zhejiang Gongshang University, Xiasha University Town, Hangzhou, China; ^3^School of Business and Economics, Knowledge, Information and Innovation, Vrije Universiteit, Amsterdam, Netherlands; ^4^Independent Researcher, Gainesville, FL, United States

**Keywords:** organizational structure, affective commitment, flexibility, opportunity exploitation, top executives, founders

## Abstract

Is flexibility or formality more useful for organizations that are pursuing improved performance? Organizational structure scholars offer opposing answers to this question, and empirical results have been mixed. Our study contributes to this research by describing a mediational model that links organizational flexibility to performance via opportunity exploitation. Specifically, we argue that flexible firms are able to exploit a greater number of opportunities, which, in turn, can improve performance. We also argue that the indirect effect of flexibility on performance via opportunity exploitation is stronger when top executives display higher affective commitment for their firms, meaning that they have a positive emotional attachment to their firms. Top executives with higher affective commitment can mitigate the downsides experienced by the staff of flexible firms, such as uncertainty and negative affect, which improves the outcomes of flexibility. Drawing on a sample of 211 firms and their founders, we find support for our hypotheses.

## Introduction

A long-running debate among organizational structure scholars has concerned the benefits of flexibility, relative to more rigid formality (Stinchcombe, 1965; Fredrickson, 1986; Adler and Borys, 1996; Pertusa-Ortega et al., 2010; Herhausen et al., 2021). Flexibility refers to a characteristic of organizations in which people’s roles exhibit high levels of variation and uncertainty, where routines are much less pervasive, and where structure can and does easily change (Adler et al., 1999; Foss et al., 2015). In contrast, formality refers to a characteristic of organizations in which people’s roles are clearly defined, patterns of interactions are routinized, and firms’ structures are stable over time. Formalization has been portrayed as beneficial to firms because it provides the structure necessary to interpret and respond to new information and it leads to production efficiencies (Jansen et al., 2005; Juillerat, 2010; Felin et al., 2012). However, flexibility has also been portrayed as beneficial to firms because it enables firms to more rapidly respond to changing customer demands and competitive conditions, redeploy resources, and produce innovative products and services (Sirmon et al., 2008; Zhou and Wu, 2010; Dibrell et al., 2014). As a consequence, the empirical record is mixed concerning the relative benefits of flexibility and formality (Herhausen et al., 2021).

The debate over the relative benefits of flexibility has unique relevance for firms that practice strategic entrepreneurship, which involves the pursuit of new market opportunities while deepening firms’ existing opportunity exploitation activities (Hitt et al., 2001; Ireland et al., 2003). Strategic entrepreneurship is a key component of firms’ ability to survive in dynamic and uncertain environments (Shimizu and Hitt, 2004; Zahra et al., 2008), and scholars have argued that elements of both flexibility and formality can contribute to firms’ survival (Eisenhardt et al., 2010; Brinckmann et al., 2019). Given the importance of the topic, researchers have extensively explored the antecedents of flexibility/formality and sought to understand the conditions that favor either greater flexibility or greater formality, such as dynamism (Sine et al., 2006; Claussen et al., 2018) characteristics of firms’ resource portfolios and capabilities (Barker and Barr, 2002; Santos-Vijande et al., 2012), and strategic orientation (Nadkarni and Narayanan, 2007).

Surprisingly, firms’ top executives have been conspicuously absent from these examinations. In particular, while some scholars have linked top executive attributes to their firms’ flexibility as an antecedent (Miller and Droge, 1986; Miller et al., 1988; Lewin and Stephens, 1994; Nadkarni and Herrmann, 2010), we have much less understanding regarding the moderating role top executives may play in facilitating the beneficial performance outcomes related to structure. This gap is critical because top executives wield enormous influence in their firms by setting the strategic vision, developing and managing firms’ behaviors, and by perceiving and responding to environmental and competitive conditions on firms’ behalf (Hambrick and Mason, 1984; Pryor et al., 2019). Top executives not only make decisions that influence their firms’ structure, such as by hiring, firing, and guiding job design (Fenton-O’Creevy, 2001; Chadwick et al., 2015), but we argue that they can also make decisions or enact behaviors that strengthen the positive relationship between structure and performance. Therefore, we focus on top executives’ affective commitment to their firms (Meyer et al., 1993; Meyer and Herscovitch, 2001). Affective commitment refers to the positive emotional attachment a top executive has with their venture, as well as their commitment to remain with it (Vandenberghe et al., 2017). Because flexibility can create stress and contribute to employee turnover (Chandler et al., 2005), top executives with a high degree of affective commitment may reduce these negative effects of flexibility because they tend to promote employees’ well-being and engage in beneficial citizenship behaviors (Meyer et al., 2002).

**FIGURE 1:**
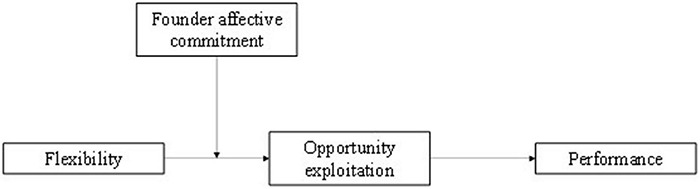
Mediation model of flexibility, opportunity exploitation, and performance, moderated by founder affective commitment.

In this paper, we draw on organizational structure and strategic entrepreneurship theories to develop and test the model presented in Figure 1. We argue that flexible organizational structure will be positively related to firms’ opportunity exploitation and that opportunity exploitation will positively influence firm performance. We also argue that the indirect effect of organizational structure on performance will be stronger when top executives exhibit higher, rather than lower, affective commitment to the firms they lead. We test our model on a sample of 211 founders and their firms in St. Petersburg, Russia.

In so doing, our study contributes to research on organizational structure, strategic entrepreneurship, and microfoundations. With regard to organizational structure research, our study is among the first to place the focus on the enabling role that top executives have on structure. To the extent that research in this field has considered the role of the top executive, it has been to focus on structure as the dependent variable and various characteristics of top executives as the predictor variables, such as need for achievement (e.g., Miller and Toulouse, 1986), locus of control, trust, ambiguity tolerance, risk propensity (Lewin and Stephens, 1994), tenure (Miller, 1991), and personality (Nadkarni and Herrmann, 2010). While this research has generated important insights, top executives’ influence may extend beyond establishing “what” structure a firm has to “how” structure influences firm outcomes. Relatedly, theoretical and empirical research on organizational structure has portrayed an unclear picture concerning structure’s outcomes. For instance, some research has found that greater levels of flexibility may be positively related to outcomes, such as innovation and performance (Worren et al., 2002; Nadkarni and Herrmann, 2010), whereas other research has found that firms that adopt a more formal structure acquire greater advantages (Sine et al., 2006; Foss et al., 2015). Yet other research has reported weak relationships between structure and performance (Pagell and Krause, 2004). One factor that may inhibit our ability to draw a clear and consistent understanding of the relationships between structure and firm-level outcomes is that the mechanisms that link structure with performance have not been fully described. This paper attempts to address this limitation by proposing a mediation model, in which structure influences opportunity exploitation, which, in turn, influences firm performance.

This paper also contributes to research on strategic entrepreneurship. How founders structure their organizations can have significant influence on how their firms perform (Colombo and Delmastro, 2008), yet few studies have explored issues related to organizational structure in firms led by their founders (Burton et al., 2019). Additionally, the pursuit of strategic entrepreneurship in a firm can place extraordinary demands on the people inside it (Ketchen et al., 2007), who have to navigate a more complex realm of organizational processes and objectives. In a more aggressively entrepreneurial firm, job demands may change, roles are ambiguous, and the future of the firm depends on riskier outcomes, which can place tremendous stress upon the people who work there (e.g., Monsen and Boss, 2009). In this paper, we argue that these stresses can be mitigated when top executives have a strong affective commitment to the firms they founded.

Finally, this paper contributes to the growing microfoundations research on top executives and how their personal characteristics can influence firm-level outcomes (Davis et al., 2009; Felin et al., 2012). Microfoundations research on organizational structure has tended to emphasize top executives’ cognition (e.g., Eisenhardt et al., 2010; Helfat and Peteraf, 2015). However, consistent with research on employees and managers (Vandenberghe et al., 2017), the emotional attachment that founders have for their firms—which can be quite passionate (e.g., Gielnik et al., 2015)—can influence firm-level outcomes as well. Our exploration of the interaction between firms’ structure and founders’ affective commitment advances our understanding of how the emotions of top executives can shape the firms they lead. In this way, we also contribute to upper echelons research, which has recently turned from examining top decision makers’ demographic characteristics to their cognition, personality, and motivation (Delgado-Garcia and De La Fuente-Sabate, 2010; Wang et al., 2016; Pryor et al., 2019).

Our paper proceeds as follows. First, we establish the theoretical and conceptual basis of the paper by describing organizational structure research as well as the characteristics of formal and flexible organizations. We next develop theoretical arguments supporting the research model presented in Figure 1. Then, we empirically test our hypotheses and describe the results. The paper concludes with a discussion of the study’s implications and limitations.

## Theory and Hypotheses

### Organizational Flexibility

An organization’s structure is composed of all of the “documented, official relationships among members of the organization” (Mintzberg, 1979, pp. 9–10). Within the umbrella of organizational structure research, scholars have explored issues related to formalization and centralization (Mansfield, 1973). Centralization, which concerns the degree to which important decisions are made at the top of a firm vs. decision-making power being dispersed throughout the firm (Egelhoff, 1988; Joseph et al., 2016), lies beyond the scope of this paper. Herein, we focus on formalization, which refers to the extent to which the roles and interactions between roles in a firm are explicitly bound by rules (Sine et al., 2006; Foss et al., 2015). Formalization represents a continuum, from firms that are highly, formalized (i.e., sclerotic bureaucracies; e.g., Hirst et al., 2011) to highly flexible (Eisenhardt and Martin, 2000). While firms can attempt to balance formalization and flexibility (Raisch and Birkinshaw, 2008), formalization and flexibility are more often portrayed as alternatives, such that firms tend to pursue either formal or flexible structure (e.g., Hempel et al., 2012; Foss et al., 2015). Scholars have offered different perspectives of formality, with some describing how formal organizational structures can strengthen firms’ strategies, pursuit of goals, and internal processes, while others have argued that formality can stifle innovative output, reduce employees’ performance, and create inefficacies due to red tape (John and Martin, 1984; Adler and Borys, 1996; Lin and Germain, 2003; Tata and Prasad, 2004).

Formalization can also affect the extent to which firms are able to exploit new opportunities. We argue that firms that exhibit greater flexibility, rather than formality, will exploit more opportunities. Opportunities are defined as “situations in which new goods, services, raw materials, markets and organizing methods can be introduced through the formation of new means, ends, or means-ends relationships” (Eckhardt and Shane, 2003, p. 336), and opportunity exploitation is defined as the “building [of] efficient, full-scale operations for products or services created by, or derived from, a business opportunity” (Choi et al., 2008, p. 335). Opportunities derive from changes in the environment, such as changing customer preferences, changing availability of resources, changing competitive conditions, and prior innovative activity (Eckhardt and Shane, 2003; Holcombe, 2003). Therefore, firms that are more flexible will have advantages over less flexible firms in exploiting new opportunities.

The first advantage flexible firms have is that their workforce tends to have greater autonomy and that the workforce can be redeployed to meet emerging needs. Employees serve as the basis of firms’ flexibility (Salvato and Vassolo, 2018). Relative to people in more formal organizations, whose roles are more rigidly determined, the people in flexible organizations may have an added degree of personal autonomy. People with greater autonomy, in turn, may be relatively more effective at developing innovative projects because fewer processes and procedures exist that can lead to pre-determined outcomes (Theurer et al., 2018). Flexible firms also have a greater ability to adjust their resource deployments, such as over the lifespan of new product development project or when encountering unexpected problems (Zhou and Wu, 2010). This means that people in a flexible firm may take on different tasks or roles over the course of a project, so that their abilities best align with what the firm demands in a situation or so that the firm can handle surprises or emerging problems (e.g., Vashdi et al., 2013). As a consequence, people in flexible firms are exposed to a greater number of more varied tasks, expanding their knowledge and experience and improving their and their firms’ learning capacity (Ortega, 2001; Li et al., 2010). This can help firms acquire and assimilate new knowledge more quickly, boosting their innovative capacity and ability to identify new opportunities and launch innovative products and services (Zahra et al., 2009).

The second advantage flexible firms have is that they may develop unique cultures and capabilities related to handling flexibility that more rigid, formal firms could lack. As Zhou and Wu (2010) note, flexible firms can become adept at reconfiguring resources to respond to dynamic environmental conditions, fostering a culture in which change and innovation is valued. When it comes to firms’ workforce, flexibility can correspond to a greater number of role changes, hires, and departures among firms’ members. The increased workforce churn can also increase heterogeneity among members (van der Vegt et al., 2010), which research has found to have a positive influence on innovation (e.g., Somech and Drach-Zahavy, 2013) as people of different knowledge, experience, and skillsets interact with each other. Finally, because greater flexibility may correspond to higher turnover (e.g., Shimizu and Hitt, 2004), flexible firms may become skilled at capturing implicit knowledge from employees, translating it into tacit knowledge, and sharing it with new members (Zellmer-Bruhn, 2003). In turn, firms with greater capacity to capture and share knowledge among its members may also be better enabled to pursue opportunity exploitation.

Certainly, excessive flexibility can come with costs, increase uncertainty, and reduce efficiencies (e.g., Eisenhardt et al., 2010). Firms that are too flexible may also be indistinguishable from very early stage startups, where basic strategic decisions have not yet been finalized, such as product/service mix or target market segment, and where basic organizational elements have not yet been established (e.g., Boeker and Wiltbank, 2005). Nevertheless, for established firms, which have set up basic organizational structure, we expect that greater flexibility will correspond with a larger number of exploited opportunities. Therefore:

*Hypothesis 1: There will be a positive relationship between firms’ flexibility and opportunity exploitation.*

### Founders’ Affective Commitment

The benefits that flexibility can bestow upon firms come with downsides, including increased employee stress and lack of integration with the firm and fellow personnel (van der Vegt et al., 2010), increased uncertainty (e.g., Gruenfeld et al., 1996), increased role ambiguity (Monsen and Boss, 2009), and less effective decision making (e.g., Juillerat, 2010). Unchecked, these factors could harm firms’ ability to function. However, we argue that when top executives have a strong affective commitment to the firms they founded, these factors are largely mitigated, which strengthens the positive relationship between flexibility and opportunity exploitation.

Affective commitment refers to the positive emotional connection a person has for an organization or another person, such as a supervisor (Meyer and Herscovitch, 2001; Vandenberghe et al., 2017). As Meyer and Allen (1991) write, a person with a strong affective commitment to their organization will “continue employment with the organization because they *want* to do so” (p. 67). Affective commitment is conceptualized as one component of an overall organizational commitment construct that also includes continuance commitment, which concerns a person’s awareness of the costs of leaving an organization, and normative commitment, which concerns the duty or moral obligation people may feel to remain with an organization (Meyer and Allen, 1991). All three components may be present in a person at the same time and motivate their behavior, and the components have also been found to interact with each other. For instance, the effects of affective commitment have been found to be stronger when coupled with high continuance commitment (e.g., Somers, 1995). All three components of organizational commitment have been found to have negative influence on employee turnover, although affective commitment has the strongest negative effect (Meyer et al., 2002). In Meyer et al. (2002) meta-analysis (see also Mathieu and Zajac, 1990), affective commitment was also found to improve job performance, organizational citizenship behaviors, and reduce absenteeism as well as stress and work-family conflict. Normative commitment was also negatively related to turnover intentions and positively related to absenteeism, job performance, and organizational citizenship behaviors. Additionally, continuance commitment was negatively associated with turnover intentions, positively associated with absenteeism, and negatively related to employee performance, while having little relationship with organizational citizenship behaviors. Research has also found empirical links between affective commitment and job satisfaction (Loi et al., 2014). Interestingly, few studies have considered the organizational commitment of firms’ top executives, focusing, instead, on how top executives may improve managers’ and employees’ commitment. Given that affective commitment is more strongly predictive of people’s behaviors, we therefore focus on how founders’ affective commitment moderates the relationship between firms’ flexibility and opportunity exploitation.

Although many founders may feel strong emotional connections to their firms (He, 2008), it is not necessarily true that all founders have high levels of affective commitment. For instance, not all founders are intrinsically motivated to launch a venture but do so because of external pressures and the necessity to support themselves by any means (e.g., Nikiforou et al., 2019). Other founders, such as portfolio entrepreneurs (e.g., Westhead et al., 2005), may have little emotional commitment to any one firm but instead treat each one as a potential, yet terminable, means to achieve financial growth.

Founders’ affective commitment can strengthen the relationship between flexibility and opportunity exploitation in a number of ways. Founders who have high affective commitment are going to be more likely to exhibit authentic leadership characteristics, meaning that they will tend to demonstrate greater care for their firms’ staff and devote time to improve their well-being (Ribeiro et al., 2020). Founders with high affective commitment are also more likely to mentor their firms’ staff (Tepper and Taylor, 2003). These tendencies might act to reduce the uncertainty people may feel related to highly flexible work environments. Additionally, founders with high affective commitment may signal to others that the firm is important to the founder and that, though the venture takes risks in pursuing new opportunities, the founder is dedicated to the overall well-being and success of the firm and the people in it, which could further reduce staff uncertainty. Research has also found that when the leaders of a firm exhibit high affective commitment, their pleasure of working can spread throughout the workforce, fostering an overall level of happiness among the staff (e.g., Sy et al., 2005; Netemeyer et al., 2010), and positive emotions have been linked to improved innovative performance (e.g., Baron and Tang, 2011).

Conversely, founders with low affective commitment do not have the same positive emotional connections to their firm, perhaps because they are treating the firm as a means to a financial end, such as portfolio entrepreneurs, or because personal circumstances compelled them to create a firm for survival (e.g., Block and Koellinger, 2009), or because the business they founded is not meeting their financial or personal expectations (Chen et al., 2017). These founders may be looking for other options outside the firm they created, which can increase the uncertainty felt by other staff members in the firm. The staff of a firm led by a founder with low affective commitment may also come to share their leader’s feelings via mood contagion (e.g., Barsade, 2002). As we argued above, founders with strong affective commitment may be able to mitigate the downsides of flexibility on their employees’ innovative output, such that flexibility has a positive relationship with opportunity exploitation. However, when founders have low affective commitment, firms’ staff members actually experience exacerbated uncertainty and more negative emotions. Staff members’ heightened uncertainty and negative emotions, when interacting with relatively flexible firm environments, can reduce firms’ innovative output and the exploitation of opportunities (e.g., Baron, 2008; Baron and Tang, 2011; Fischer et al., 2019). Therefore, we offer the following hypothesis:

*Hypothesis 2: The relationship between flexibility and opportunity exploitation will be stronger for firms led by founders with higher affective commitment.*

### Opportunity Exploitation and Performance

We further predict that firms that exploit a greater number of opportunities by introducing innovative new products and services will also tend to achieve better performance (Rosenbusch et al., 2011). The foundation of strategic entrepreneurship is that firms’ environments are uncertain and dynamic (Hitt et al., 2001), which creates market disequilibria that constitute opportunities (Eckhardt and Shane, 2003). Firms that are able to identify these disequilibria and act on them—such as by producing new products, methods of production, entering new markets, or other forms of innovation (Schumpeter, 1934)—will be able to secure momentary rents through organizing and deploying resources to exploit opportunities. So long as firms are able to protect their unique knowledge or their right to exploit an opportunity (e.g., via patenting, Alvarez and Barney, 2004), they will have the ability to derive financial value from it. Firms may also benefit indirectly from opportunity exploitation in a number of ways (Rosenbusch et al., 2011). The research and development necessary to exploit opportunities may lead to the generation of new knowledge in firms, which is itself a resource (Van de Ven and Polley, 1992), as well as develop absorptive capacity, which constitutes firms’ ability to acquire new knowledge and disseminate it throughout the organization (Cohen and Levinthal, 1990). Rosenbusch et al. (2011) also describe how the ability to perceive and adapt to changing customer preferences, competitor actions, and environmental dynamics via opportunity exploitation may help firms develop stronger dynamic capabilities, which refer to an overall ability to effectively adapt to firms’ external conditions (Eisenhardt and Martin, 2000; Siren et al., 2012). Alternatively, firms that exploit fewer opportunities may become detached from market conditions because they focus on meeting the needs of current customers rather than on identifying new opportunities (Crossan et al., 1999). Over time, the value they obtain from existing opportunities will likely be lost through imitative competition or increasing resource prices (Eckhardt and Shane, 2003). Therefore, we hypothesize:

*Hypothesis 3: There will be a positive relationship between opportunity exploitation and performance.*

### Indirect and Conditional Indirect Effects

Flexibility consists of firms’ capacity to adjust their workforces in response to changing environmental conditions. We argue that greater flexibility enables firms to exploit a greater number of opportunities. Relative to firms that exhibit greater formality, in which roles and interpersonal relationships are more rigidly established, flexible firms will have greater ability to realign people’s efforts to correspond with the most important tasks throughout the life of innovative projects. Flexible firms may also have greater learning capacity than more formalized firms, and their workforces will display greater levels of heterogeneity, both of which have been found to positively affect firms’ innovative outputs and consequent ability to exploit opportunities. In turn, opportunity exploitation can strengthen firms’ performance directly because it enables them to capture new value that arises from market disequilibria and indirectly because it strengthens firms’ knowledge base as well as enhancing firms’ absorptive capacity. Therefore:

*Hypothesis 4: Flexibility will have a positive indirect effect on firm performance via opportunity exploitation.*

We also propose that the indirect effect of flexibility on performance via opportunity exploitation will be stronger for founders who have higher affective commitment to their firms. Founders with high affective commitment enjoy working for their firms for the sake of the work, and this enjoyment can be transmitted to other staff members, which strengthens their creativity (Baron and Tang, 2011). Additionally, while staff in flexible firms may experience higher levels of uncertainty, which can harm their ability to perform, this uncertainty can be partly mitigated when founders demonstrate a strong affective commitment to the firm. For these reasons, we propose that the relationship between flexibility and opportunity exploitation will be stronger when founders have higher affective commitment. As a consequence, we expect these firms to also achieve higher performance due to their enhanced opportunity exploitation. In contrast, founders who have low affective commitment to their firms will likely display less pleasure or even displeasure working in their firms, which can negatively influence staff affect (e.g., Sy et al., 2005) reducing their innovative and creative performance. Additionally, founders with low affective commitment will also be less effective at mitigating the uncertainty staff members experience concerning their firms’ future and their roles in them. Staff members’ heightened uncertainty can also negatively influence firms’ creative opportunity exploitation activities (Zhang and Zhou, 2014). Lower opportunity exploitation can eventually result in lower performance outcomes for firms led by founders with low affective commitment. Therefore, we hypothesize:

*Hypothesis 5: The indirect effect of flexibility on performance via opportunity exploitation will be stronger for firms led by founders with higher affective commitment.*

## Materials and Methods

### Sample and Procedure

To test our hypotheses, we collected data from a sample of 211 entrepreneurs who founded businesses in St. Petersburg, Russia. Participants were, on average, 30.69 years old (*SD* = 6.65) and about 67% were female. Most participants (81.52%) had obtained a bachelor’s degree, and 4.27% had completed post-graduate education. They represented firms that were, on average, 9.47 (*SD* = 2.64) years old and employed 12.97 (*SD* = 18.78) people. The founders in our sample were younger and were more often female and the businesses were smaller when compared to samples of other studies conducted in Russia (e.g., Pissarides et al., 2003; Seawright et al., 2008). The firms came from a variety of industries, including retail, computer and IT services, bars and restaurants, tourism, and business-to-business services.

Participants were recruited using a snowball sampling procedure (e.g., Grant and Mayer, 2009; Ong et al., 2018). To recruit participants, emails and social media contacts were sent to founders who were affiliated with a Russian graduate business school, whether as alumni or had participated in the school’s programs as speakers, judges, or mentors. Participants were sought (and retained) if they were their firm’s founder and were currently the top decision makers of their firms. Founders who indicated they could not participate in the study were invited to provide names of other founders. In sum, 348 entrepreneurs were contacted and 211 participated, which represents a 60.63% response rate. Using the power function in Stata 14, with an estimated model r-squared of 0.15 and a probability of error of 0.05, we determined that our sample size was sufficient.

Participants were asked to respond to surveys in face-to-face interviews, which were conducted by members of the research team. Before the surveys were administered, they were written in English, translated into Russian, and back translated into English by a graduate student otherwise unaffiliated with the project. Any discrepancies between the translations were resolved. Due to the potential that common method bias could influence our results, we also split data collection into two phases (Podsakoff et al., 2003). In the first phase, we collected the control variables as well as the independent and moderator variables. In the second phase, we collected data for the mediator and dependent variables. First phase and second phase interviews were completed 1 month apart.

### Measures

#### Flexibility

Flexibility was measured using two items: “In the last 2 months, to what extent did your team add new roles,” and “In the last 2 months, to what extend did your team change which team members held each roles?” Participants responded to these items via a 7-point Likert type scale (1 = not at all, 7 = to a great extent). The Cronbach’s alpha for these items is 0.67.

#### Affective Commitment

Affective commitment was measured using seven items developed by Porter et al. (1974). Example items included: “I would be happy to spend the rest of my career with my venture,” “I really feel as if my venture’s problems are my own,” and “My venture has a great deal of personal meaning for me.” Participants responded to these items via a 7-point Likert type scale (1 = strongly disagree, 7 = strongly agree). The Cronbach’s alpha for these items was 0.75.

#### Opportunity Exploitation

Opportunity exploitation was measured with two items: “In the last year, to what extent did you introduce new products and services,” and “In the last year, to what extent did you enter new markets?” Participants responded to these items via a 7-point Likert type scale (1 = not at all, 7 = to a great extent). This measure is similar to others used in prior research (e.g., Barney et al., 2018), which have been used to identify how many new opportunities to create customer value firms have identified and pursued. Because our emphasis in this study is on the production of new products and services and new market entry, our items attempt to assess the actual behaviors founders enacted to create value. The Cronbach’s alpha for these items is 0.73.

#### Performance

Firm performance was measured using 11 items developed by Delaney and Huselid (1996). Objective financial performance can be difficult to obtain for private firms, such as those represented by the participants in our study; moreover, given these firms are relatively young startups, typical financial performance measures may not reflect relevant performance metrics (Rompho, 2018). Therefore, we relied on Delaney and Huselid’s (1996) measure to assess founders’ subjective performance, using items that ask participants to evaluate their firms’ performance over the past 3 years relative to competitors along various dimensions, using a 7-point Likert type scale (1 = much worse; 7 = much better). Dimensions included: satisfaction of customers and clients, relations among employees, growth in sales, profitability, and market share. In prior research, subjective performance assessments have been found to have high correlation with objective performance (Wang et al., 2011). The Cronbach’s alpha for the items used in our study was 0.79.

#### Controls

We included controls for founders’ age, gender, and education level because these attributes can influence the aggressiveness with which firms pursue and exploit new opportunities and how they may evaluate their performance outcomes. For instance, age has been found to be negatively associated with risk taking (Mata et al., 2016), while age and education can reflect founders’ greater experience, which can give them an advantage when identifying new opportunities (e.g., Gregoire et al., 2010).

Because our performance measure was subjective, we believed it was important to control for founders’ satisfaction with their firms’ performance, which could affect their assessments. We used four items developed by Gruber et al. (2010) to measure founders’ performance satisfaction. These items included: “We are very satisfied with the development of our business in comparison with other firms in our industry,” and “We are very satisfied with our growth rate in comparison with our strongest competitors.” The Cronbach’s alpha for these items was 0.82.

We also included controls for firm age and firm size (i.e., number of employees) because these characteristics may correspond with firms’ resource portfolios, such that older, larger firms tend to possess greater resources than younger firms (Thornhill and Amit, 2003).

#### Common Method Bias

We collected data via face-to-face surveys, and participants’ responses were used to build measures for the study’s constructs. Therefore, common method bias can potentially influence our analyses (Podsakoff et al., 2003). Specifically, social desirability bias—that is, participants’ desire to appear in a more positive light—may lead participants to overestimate their firms’ performance, opportunity exploitation, or their own affective commitment. There may also be measurement context effects, another form of common method bias, because the same person administered the face-to-face surveys. We followed two techniques recommended by Podsakoff et al. (2003) to mitigate the potential for common method bias to influence our results. First, data collection was separated into two phases, so that the dependent variable was not captured at the same time as the control, independent, and moderator variables. Second, we employed a statistical technique, which uses a common latent variable, to control for the influence of common method bias. To produce this common latent variable, we used structural equation modeling to calculate a latent factor variable using the multi-item, non-binary measures (i.e., flexibility, affective commitment, and performance). This common latent variable was included as a control variable in our regression results, and can account for common method bias (Podsakoff et al., 2003). The inclusion of this variable in our analyses did not influence our results. Additionally, we find significant interaction effects (i.e., Hypothesis 2 is supported). Common method bias has been found to suppress interaction terms (Siemsen et al., 2010). Because we find support for Hypothesis 2, common method bias may not be a serious threat to our analyses.

### Analytic Strategy

We used the gsem function in Stata 14 to specific a path model to test our hypotheses. Variables included in the interaction term were first mean centered. Robust standard errors were used, given the presence of non-normal and heteroskedastic residuals. To specify the model, we estimated paths between firms’ flexibility and opportunity exploitation and between opportunity exploitation and performance. A path was also specified from the interaction of flexibility with affective commitment on opportunity exploitation. Paths were specified from the control variables onto opportunity exploitation and onto performance. Paths were also specified from both flexibility and affective commitment onto performance. In order to test the indirect effect of flexibility on performance via opportunity exploitation, we calculated bias-corrected confidence intervals using bootstrapping (5,000 repetitions) (MacKinnon et al., 2004). To test the moderated mediation hypothesis, we employed a technique described by Hayes (2018), which uses bootstrapping to produce bias-corrected confidence intervals for the indirect effect of role fluidity on performance via opportunity exploitation at two levels of the moderator (± 1 SD).

The means, standard deviations, and correlations are reported in Table 1.

**TABLE 1:** Means, standard deviations, and correlations.

	Mean	*SD*	1	2	3	4	5	6	7	8	9
1. Entrepreneur age	30.69	6.65									
2. Entrepreneur gender	0.67	0.47	–0.05								
3. Entrepreneur education	3.85	0.58	−0.21**	−0.17*							
4. Performance satisfaction	4.51	1.20	−0.15*	–0.13	–0.06						
5. Firm age	9.47	2.64	–0.09	0.13	–0.02	–0.13					
6. Firm size	12.97	18.78	0.13	–0.12	0.00	0.15*	−0.32**				
7. Flexibility	3.16	1.92	−0.17*	0.02	0.02	0.17*	–0.03	0.09			
8. Affective commitment	2.32	0.93	–0.07	–0.03	0.03	0.23**	–0.05	0.00	0.06		
9. Opportunity exploitation	3.80	1.92	–0.11	0.09	–0.03	0.21**	–0.08	0.09	0.23**	0.27**	
10. Performance	4.84	0.76	0.02	−0.19**	–0.11	0.67**	−0.18**	0.25**	0.15*	0.30**	0.31**

N = 211; *p < 0.05, **p < 0.01.

## Results

**FIGURE 2:**
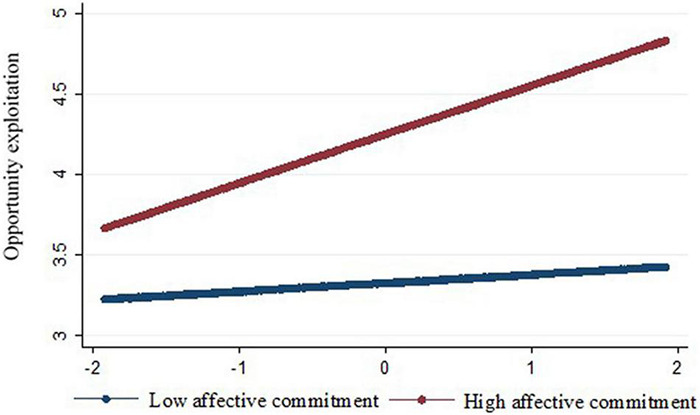
Interaction of flexibility and founder affective commitment on opportunity exploitation.

The results of the path analysis are reported in Table 2. Hypothesis 1, which predicted that flexibility would be positively related to opportunity exploitation, was supported (*B* = 0.22, *p* < 0.01). Hypothesis 2 predicted that opportunity exploitation would be positively related to performance. This hypothesis was supported (*B* = 0.06, *p* < 0.001). Hypothesis 3 predicted that flexibility would have a positive indirect effect on performance, via opportunity exploitation. Table 3 includes the results for our indirect and conditional indirect effects. Our results support this hypothesis, given that the confidence interval for the indirect effect does not include zero [indirect effect = 0.03, 95% bias-corrected CI: (0.012, 0.053)]. Hypothesis 4 predicted that the relationship between flexibility and opportunity exploitation would be stronger when founders’ affective commitment to their firms was higher. This hypothesis was supported (*B* = 0.16, *p* < 0.01). The interaction is plotted in Figure 2, with the relationship between flexibility and opportunity exploitation plotted for entrepreneurs who are low (–1 SD) and high (+ 1 SD) on affective commitment. For founders who are low on affective commitment, there is no relationship between flexibility and opportunity exploitation (simple slope = 0.07, *p* > 0.05); whereas, for founders who are high on affective commitment, the relationship is positive (simple slope = 0.36, *p* < 0.001). Therefore, our evidence indicates that founders who have a higher affective commitment to their firm are able to leverage higher rates of flexibility to benefit opportunity exploitation efforts whereas founders with lower affective commitment are not. Hypothesis 5 predicted that the indirect effect of flexibility on performance via opportunity exploitation would be stronger for founders with higher, rather than lower, affective commitment. Bootstrapped moderated mediation results are reported in Table 3, which indicates that the hypothesis is supported [index of moderated mediation = 0.01, 95% bias-corrected CI: (0.001,0.018)]. Founders who had higher affective commitment to their firms achieved better performance via flexibility’s indirect effect through opportunity exploitation [conditional indirect effect = 0.04, 95% bias-corrected CI: (0.015, 0.064)]. Interestingly, founders who had lower affective commitment also acquired a performance benefit from the indirect effect of flexibility [conditional indirect effect = 0.02, 95% bias-corrected CI(0.007, 0.044)]. However, the conditional indirect effect was significantly stronger when founders had higher affective commitment [Δ conditional indirect effect = 0.01, 95% bias-correct CI(0.003, 0.034)].

**TABLE 2:** Path analysis.

	Opportunity exploitation		Performance	
	*B*	Robust Std. Err.	B	Robust Std. Err.
Constant	76.97	110.34	–3.63	26.61
Common latent variable	0.04	0.27	0.78***	
Entrepreneur age	–0.02	0.02	0.01	0.00
Entrepreneur gender	0.47	0.28	–0.07	0.07
Entrepreneur education	–0.06	0.21	–0.07	0.07
Performance satisfaction	0.20	0.12	0.16***	0.03
Firm age	–0.04	0.05	0.00	0.01
Firm size	0.07	0.06	0.03***	0.00
Flexibility (H1)	0.22**	0.08	–0.01	0.02
Affective commitment	0.49***	0.11	0.11**	0.05
Flexibility * Affective commitment (H4)	0.16**	0.06		
Opportunity exploitation (H2)			0.06***	0.02
*R* ^2^	0.17		0.74	

N = 211; *p < 0.05, **p < 0.01, ***p < 0.001.

**TABLE 3:** Indirect effects of flexibility on performance.

	*B*	Bootstrapped Std. Err.	Lower 95% CI	Upper 95% CI
Unmoderated indirect effect (H3)	0.03	0.01	0.012	0.053
Index of moderated mediation (H5)	0.01	0.00	0.001	0.018
Conditional indirect effect, high affective commitment	0.04	0.01	0.015	0.064
Conditional indirect effect, low affective commitment	0.02	0.01	0.007	0.044
Difference of conditional indirect effects	0.01	0.01	0.003	0.034

*N = 211.*

## Discussion

Does formality or flexibility benefit firms more when it comes to opportunity exploitation and the pursuit of improved performance? Researchers have offered opposing views on this question, with some arguing that formality provides the structural basis that enables firms to operate efficiently and to quickly assimilate new information (e.g., Foss et al., 2015) while others have argued that flexibility can enable firms to more rapidly redeploy resources, assign people to new tasks to meet new demands, and foster a culture of exploration (e.g., Zhou and Wu, 2010; Vashdi et al., 2013). Our findings address this debate in several ways.

First, we find that, for our sample of founder-led firms in St. Petersburg, Russia, that flexibility does improve firms’ opportunity exploitation. This finding is consistent with entrepreneurship research, which describes how opportunities emerge from changes in firms’ environments (Holcombe, 2003), and how firms that have the capacity to adapt to these environmental changes may able to exploit a greater number of them as opportunities (e.g., Bock et al., 2012). Second, we find that this relationship is stronger for firms that are led by founders with high levels of affective commitment. Such founders have a strong, positive emotional connection to the firms they created, which contributes to their positive affect in the workplace, mentorship efforts, tendency to display authentic leadership, and helping others perform their jobs (e.g., Tepper and Taylor, 2003; Sy et al., 2005; Ribeiro et al., 2020). We also argue that founders with high affective commitment may reduce the uncertainty experienced by other staff members of the firm because the founder is clearly emotionally invested in the long-term viability of the firm. This might be especially important for staff members of firms that are engaged in strategic entrepreneurship—the outcomes of which are uncertain and could put firms at risk—because these members may experience heightened levels of uncertainty (e.g., Monsen and Boss, 2009). Third, we find that flexibility has an indirect effect on performance via opportunity exploitation and that this effect is stronger when founders have higher, rather than lower, levels of affective commitment (However, interestingly, our results show that the indirect effect of flexibility is significantly positive, whether founders have higher or lower levels of affective commitment).

### Research Implications

Our study may have several implications for research on organizational structure, strategic entrepreneurship, and microfoundations. Prior research on the role of the top executive and organizational structure has tended to use top executive characteristics to predict levels of firms’ flexibility (e.g., Miller and Toulouse, 1986; Nadkarni and Herrmann, 2010). However, in our paper, we focus on the relatively unexplored question concerning how top executives’ characteristics might influence the effectiveness of organizational structure. Top executives—especially founder top executives—have considerable ability to influence the strategic direction, resources, and behaviors within their firms (Hambrick and Mason, 1984; Pryor et al., 2019). And founders may have even greater influence, given that they are often owners of the firms they lead, that they have played a personal role in the creation and management of the firm since its inception, and that they bear extensive financial and personal risks if their firms fail (Gedajlovic et al., 2004; Souder et al., 2012; Yamakawa et al., 2015). Therefore, while top executives and the founders we examine in our study have the ability to determine firms’ structure, it may also be worthwhile to examine how top executives can rely on existing firm structure to leverage improved firm outcomes. Because strategic entrepreneurship is rife with uncertainty and because flexibility may add to staff members’ stress (e.g., Monsen and Boss, 2009), we focus on the moderating effect of founders’ affective commitment, which prior research has found to be related to more satisfied, better performing employees (e.g., Michaelis et al., 2009; Jin et al., 2016). However, other top executive attributes may also affect the effectiveness of firm structure, which future research could explore. Additionally, while we focus on the sole founding top executive, many firms are lead by teams of top managers, and understanding how team attributes can influence the effectiveness of organizational structure may also prove to be a fruitful path of research.

This study may also contribute to organizational structure research by presenting a mediational mechanism by which flexibility influences firm performance. Existing research has presented a mixed view concerning the relative benefits of flexibility or formality. For instance, some research has found that formality tends to promote innovation in firms (e.g., Sine et al., 2006; Foss et al., 2015), while other research argues that flexibility can promote innovation (e.g., Worren et al., 2002; Nadkarni and Herrmann, 2010). Additionally, research has produced inconsistent findings regarding the flexibility-performance relationship, which suggests that if flexibility influences performance, it may do so via mediational mechanisms (Herhausen et al., 2021). In particular, flexibility may enable firms to exploit new opportunities by developing new products and services to meet customer needs (Shimizu and Hitt, 2004), so we tested a model in which opportunity exploitation mediated the effect of flexibility on performance. [Consistent with prior research, we found no relationship between flexibility and performance (*B* = 0.01, *p* > 0.05)]. In this way, we hope our study advances our understanding of the mechanisms that link flexibility with performance.

We also contribute to research on strategic entrepreneurship and entrepreneurship more broadly. Researchers have previously acknowledged that entrepreneurship, whether in new or existing firms, can be difficult and stressful for the members of those firms (Ketchen et al., 2007; Monsen and Boss, 2009). The exploration for new opportunities to exploit can be challenging for firms’ employees because it creates uncertainty regarding their roles (Rafferty and Griffin, 2006; Andersen, 2017). However, little research has explored the consequences of flexibility and entrepreneurship on the well-being and performance of firms’ employees, and even fewer studies have empirically explored how top executives can mitigate the more harmful downsides of entrepreneurship. In our research, we find that top executives who care more deeply about their firms and enjoy their work might be able to reduce any negative consequences felt by employees in flexible, entrepreneurially oriented firms. Additionally, the topic of organizational structure has only recently begun to attract attention from entrepreneurship scholars (DeSantola and Gulati, 2017; Burton et al., 2019). We hope our work, which relies on a sample of firms that are relatively young—fewer than 10 years old, on average—may help advance this burgeoning stream of research.

Finally, we hope to contribute to microfoundations research. Over the past decade, scholars have dedicated increasing attention to understanding the individual- or group-level antecedents of firm-level phenomena (Davis et al., 2009), and significant progress has been achieved in developing our understanding of how firms’ capabilities and other behaviors are influenced by the people who lead or work in firms (Felin et al., 2015). In this vein, top executives’ cognition, personality, and motivations have received scholars’ attention (e.g., Helfat and Peteraf, 2015; Wang et al., 2016; Pryor et al., 2019). However, scholars have called for greater attention to top executives’ emotions (Bromiley and Rau, 2016). Therefore, this study may advance our understanding—and build the empirical record—concerning one aspect of top executives’ emotions (i.e., affective commitment).

### Practical Implications

Our study may also have several practical implications. The first is that flexibility does appear to benefit firms’ opportunity exploitation efforts, and that through exploiting a greater number of opportunities, firms achieve performance advantages. Recent research has found that firms that avoid typical planning procedures and practice organizational improvisation tend to discover more entrepreneurial opportunities (Fultz and Hmieleski, 2021). Organizational improvisation is defined as the “deliberate and extemporaneous composition and execution of novel action” performed by and among people in an organization (Fultz and Hmieleski, 2021, p. 4), and it promotes interactions between people, which can lead to unexpected new ideas, as well as resource recombinations, which can foster innovation. When taken together with our findings that flexibility—firms’ ability to move people to different roles to accomplish different tasks—promotes opportunity exploitation, top executives may consider adjusting their practices to foster improvisation. For instance, the practice of job rotation, or giving employees several responsibilities in the firm among which they frequently switch (Cosgel and Miceli, 1999), is often planned, with managers relying on decision criteria to make new assignments (e.g., Jorgensen et al., 2005). Instead, incorporating randomness, in terms of the kinds of jobs employees can be rotated to or to the rotation schedule, might encourage an even higher level of innovative output in a firm because it maximizes serendipity (De Rond, 2014).

A second practical implication is that flexibility and the pursuit of innovation and strategic entrepreneurship introduces uncertainty in the firm, which can harm employees’ satisfaction (e.g., Monsen and Boss, 2009; De Clercq et al., 2016) and can even terrify them (Meijers, 2002). Top executives may consider adopting practices that can reduce these negative, unintended consequences. For instance, De Clercq et al. (2016) suggest that knowledge sharing and emphasizing friendships among employees may be able to offset the downsides of more rigorous job demands. This advice may be especially true for top executives who have low affective commitment to their firms. These top executives, which may include serial entrepreneurs or entrepreneurs of venture-capital backed firms who have personal exit strategies for their firms, may consider avoiding transformational leadership approaches, the success of which depend on top executives’ authenticity (Spitzmuller and Ilies, 2010), in favor of more transactional approaches, which are resilient to top executives’ commitment to the firm (e.g., Deichmann and Stam, 2015). For example, top executives could make employee rewards explicitly connected to top executives’ successful exit, such as by providing cash payouts for employees should the firm experience a successful acquisition or initial public offering.

### Limitations

Finally, this study has a number of limitations. First, our sample consists of founding top executives of businesses in Russia, which may have unique characteristics that reduce the generalizability of our findings. Second, as we argue, founders have several distinguishing attributes from hired executives, such as greater legitimacy, status, ownership, and experience in their firms (e.g., Gedajlovic et al., 2004; Pryor et al., 2019), which could suggest that founders may have greater influence in their firms than hired executives. Future research could explore this issue by comparing samples of founders and non-founders, the differences in their affective commitment for their firms, and how affective commitment interacts with flexibility or other firm attributes to influence firm performance. Third, in developing our moderation hypotheses, we largely rely on existing theory and empirical research to explain how founders’ affective commitment can strengthen the relationship between flexibility and opportunity exploitation, and our explanation concerns how affective commitment can reduce some of the negative consequences felt by the staff of flexible firms. However, we do not directly measure the effects of flexibility on firms’ staff. Future research may explore these consequences in order to establish a fuller understanding of the mediational processes that link flexibility to performance. Fourth, our measure of opportunity exploitation may not account for differences in the innovativeness of the products and services firms in our sample are producing. These products and services may be incrementally or radically innovative, which could have implications for firms’ performance and the importance of flexibility (e.g., Chang et al., 2014).

Nevertheless, we hope that our paper, which turns the focus on how top executives’ attributes can moderate the effect of flexibility on opportunity exploitation and performance, contributes to organizational structure research. While scholars have more extensively studied the relationship between top executives and organizational structure, the mechanisms by which top executives can influence the effectiveness of organizational structure have received much less attention. In particular, we find that when top executives of flexible firms enjoy their work, their firms tend to launch more products and services, which contributes to firms’ overall performance. Meanwhile, flexibility was found to have no direct effect on performance, suggesting that mediational mechanisms may help drive the benefits related to flexibility.

## Data Availability Statement

The raw data supporting the conclusions of this article will be made available by the authors, without undue reservation.

## Ethics Statement

The studies involving human participants were reviewed and approved by the OSU Human Subjects Research Office, Oklahoma State University. The patients/participants provided their written informed consent to participate in this study.

## Author Contributions

CP analyzed the data and wrote the initial draft. AS and IP collected the data. AS, IP, and CL assisted with manuscript revisions. All authors contributed to the article and approved the submitted version.

## Conflict of Interest

The authors declare that the research was conducted in the absence of any commercial or financial relationships that could be construed as a potential conflict of interest.

## Publisher’s Note

All claims expressed in this article are solely those of the authors and do not necessarily represent those of their affiliated organizations, or those of the publisher, the editors and the reviewers. Any product that may be evaluated in this article, or claim that may be made by its manufacturer, is not guaranteed or endorsed by the publisher.
